# Long-term Effects of Multiple Glucocorticoid Exposures in Neonatal Mice

**DOI:** 10.3390/behavsci1010004

**Published:** 2011-12-30

**Authors:** Susan E. Maloney, Kevin K. Noguchi, David F. Wozniak, Stephen C. Fowler, Nuri B. Farber

**Affiliations:** 1 Department of Psychiatry, Washington University School of Medicine, St. Louis, MO 63110, USA; E-Mails: maloneys@psychiatry.wustl.edu (S.E.M.); noguchik@psychiatry.wustl.edu (K.K.N.); farbern@psychiatry.wustl.edu (N.B.F.); 2 Department of Pharmacology and Toxicology and Life Span Institute, University of Kansas, Lawrence, KS 66045, USA; E-Mail: scfowler@ku.edu; 3 Department of Psychiatry, Box 8134, 660 S. Euclid Ave., Washington University School of Medicine, St. Louis, MO 63110, USA

**Keywords:** glucocorticoid, dexamethasone, neuromotor deficits, motor co-ordination, complex activity wheel, cerebellum, internal granule layer, neuron loss, apoptotic cell death

## Abstract

Glucocorticoids (GCs) such as dexamethasone (DEX) or betamethasone are repeatedly administered for up to a month to prematurely born infants as a treatment for chronic lung dysfunction. Results of clinical trials have shown that the use of GCs in these infants induces long-term deficits in neuromotor function and cognition. We have previously shown that a single exposure to clinically relevant doses of DEX or other GCs in the mouse during a period corresponding to the human perinatal period produces a dramatic increase in apoptotic cell death of neural progenitor cells in the developing cerebellum. To provide a model approximating more chronic clinical dosing regimens, we evaluated possible behavioral effects resulting from repeated exposures to DEX and subsequent GC-induced neuronal loss where neonatal mouse pups were injected with 3.0 mg/kg DEX or saline on postnatal days 7, 9, and 11 (DEX3 treatment). Adult, DEX3-treated mice exhibited long-term, possibly permanent, neuromotor deficits on a complex activity wheel task, which requires higher-order motor co-ordination skills. DEX3 mice exhibited impaired performance on this task relative to saline controls in each of two independent studies involving separate cohorts of mice. Histopathology studies utilizing stereological neuronal counts conducted in behaviorally-tested mice showed that the DEX3 treatment resulted in a significant decrease in the number of neurons in the internal granule layer (IGL) of the cerebellum, although the number of neurons in the Purkinje cell layer were unchanged. The results suggest that multiple neonatal DEX exposures can produce chronic deficits in fine motor co-ordination that are associated with cerebellar IGL neuronal loss.

## 1. Introduction

There is currently much controversy regarding the clinical use of glucocorticoids (GCs) for the treatment of respiratory dysfunction associated with premature birth [1]. Much of this concern has arisen after several placebo-controlled double blind clinical studies have shown that the chronic postnatal use of GC therapy can cause permanent neuromotor and cognitive deficits [2,3]. Consequently, it has been recommended that postnatal GC therapy not be used outside of placebo controlled clinical studies [2]. Thus, it is of great concern that an estimated 10% of prematurely born infants still receive this treatment [4]. In addition, while a single prenatal exposure to GCs is generally considered safe [5], more recent research has found that multiple GC treatments can produce decreased head circumference (a proxy measurement for brain size), length, and weight in neonates at birth [6]. Overall it is estimated that approximately 7–10% of all pregnant women receive prenatal GC therapy [7]. Taken as a whole, despite the large percentage of humans exposed to this therapy and the mounting concerns regarding its safety, surprisingly little is known about how GC exposure can produce neuropathological sequelae and long-term behavioral deficits.

In previous research [8,9] we established that both acute and chronic GC exposure produces neural progenitor cell (NPC) apoptosis in the external granule layer (EGL) of the developing mouse cerebellum. This toxicity occurs at clinically relevant doses and has an equivalent human window of vulnerability that would include all ages during which perinatal GC therapy would be used—from 20 weeks of gestation to 6.5 weeks after birth [8]. The EGL is a transient proliferative layer located in the outermost portion of the immature cerebellum and is solely responsible for the production of the granule cell neurons of the internal granule cell layer (IGL). The amount of neurogenesis in the EGL is quite extensive and leads to the production of a homogenous population of neurons in the IGL so numerous they represent over half the neurons in the entire brain [10–13]. Once its job of producing new neurons is complete, the EGL rapidly disappears around the second week of life in rodents. Based on this information, it is not surprising that the premature loss of NPCs due to GC-induced apoptosis leads to permanent decreases in the number of cerebellar internal granule layer neurons [8].

In previous rodent research, we presented preliminary evidence that a one-time neonatal exposure to dexamethasone (DEX) produced long-term deficits in neuromotor function [8]. Since clinical research suggests multiple exposures to GCs are more harmful than a single acute dose and multiple exposure regimens are still currently in use, both pre-and post-natally [5,6,14,15], we sought to extend our initial findings by subjecting neonatal mice to multiple exposures of DEX and then by examining the long-term behavioral and histological effects. In order to better characterize the neuromotor functional deficits we also employed a more extensive battery of behavioral tests.

## 2. Materials and Methods

### 2.1. Experimental Design

The typical range of doses for DEX used in rodent research to probe the glucocorticoid system is 1–10 mg/kg [16–19]. In our previous work [8] we found DEX, 3.0 mg/kg on postnatal day (PND) 7, produced mild deficits in vertical rearings but had no effects on other activity-related variables. We, therefore, chose this dose for our subsequent behavioral work and elected to start our initial exposure on PND 7. Two studies were conducted on independent cohorts of naive mice to evaluate the behavioral and histopathological effects of exposing neonatal mice to DEX on 3 different PNDs. DEX, when given chronically, can retard growth, an effect commonly referred to as “stunting”. An every other day exposure of DEX was used instead of a daily exposure in order to limit weight loss or growth inhibition. Based upon these considerations, neonatal mice were treated with either saline or DEX (3.0 mg/kg) on PNDs 7, 9 and 11 (DEX3 treatment), and then their behavioral performance on several sensorimotor-based tests was studied beginning in the early post-weaning period and into early adulthood (Table 1). In the first study (Study 1), a 1-h locomotor activity/exploratory behavior test was administered to the mice on the day of weaning (PND 21), followed 5 days later by an assessment on a battery of sensorimotor measures over 2 days designed to assess strength, balance and coordination. Mice were tested on the rotarod beginning the day after completing the sensorimotor battery to evaluate more complex motor coordination skills that may be compromised in mice with cerebellar neuropathology. Forelimb grip strength was also assessed approximately one and a half months later to determine if decreased strength might have affected performance on any of the sensorimotor measures. Ten days following completion of grip strength testing the mice were evaluated on a modified version of the motor skill sequence procedure described by Liebetanz and colleagues [20], which requires a high degree of central motor co-ordination abilities to execute finely co-ordinated movements between the forelimbs and hindlimbs.

**Table 1:** Tests to evaluate behavioral effects of multiple neonatal DEX exposures.

**Behavioral Tests**	**Ages At Testing (postnatal days; PND)** (Study 1/Study 2)
1-h Locomotor	PND 21/PND 21
Sensorimotor Battery	PND 25/PND 26
Rotarod	PND 28/PND 29
Grip Strength	PND 74/PND 34
Contact Righting	Not Done/PND 41
Complex Activity Wheel	PND 88/75

In an effort to replicate the behavioral results from Study 1, a second study (Study 2) was conducted on an independent cohort of mice using a similar experimental design (Table 1), although additional studies were included to evaluate the acute and long-term histopathological effects of neonatal DEX exposure. One additional component involved using a separate group of mice that received a single exposure to DEX (3.0 mg/kg) or saline on PND 7 and were euthanized 4 h later to demonstrate and illustrate the acute histopathological effects of DEX to induce rapid and selective apoptotic cell death in proliferating neural progenitor cells in the external granule layer of the cerebellum. The procedures and scheduling of behavioral testing in Study 2 were similar to those used in Study 1 except that mice were also evaluated on a contact righting test to assess whether there were any gross vestibular disturbances in DEX3 mice that might affect sensorimotor performance. In addition, the grip strength test was administered on PND 34 directly after the rotarod test instead of on PND 75 as it was in Study 1. Forelimb grip strength was measured at an earlier age in Study 2 since the DEX3 mice exhibited impaired performance on the inverted screen in Study 1 and testing the mice shortly after completing the sensorimotor battery would provide information on whether impaired grip strength was at least partially responsible for the inverted screen deficits. It also provided us with data on grip strength during both juvenile and adult ages of the DEX3 mice. After the completion of behavioral testing, the brains of these mice were studied histologically to estimate the number of surviving neurons in the granule and Purkinje cell layers.

### 2.2. Animals and Dexamethasone Treatment

All animal care procedures and experimental protocols were approved by the Washington University in St. Louis Animal Studies Committee. For Study 1, 5 litters produced from pregnant C57BL/6 dams (Harlan, IN, USA) were culled to 6 pups per litter to equalize maternal care across litters. Upon weaning, the mice were housed in groups of littermates in translucent plastic cages measuring 28.5 cm × 17.5 cm × 12 cm. The same procedures were conducted on 7 litters produced from pregnant C57BL/6 dams for Study 2. Upon weaning, the mice were group housed, using random distribution of treatment groups across cages. Standard lab diet and water were available *ad libitum* throughout the studies. Colony room lighting was maintained on a 12:12 hour light/dark cycle, with room temperature (∼20–22 °C) and relative humidity (50%) being controlled automatically.

In Study 1, pups received an intraperitoneal injection of 3.0 mg/kg DEX (n = 15; 6 females, 9 males) on each of PNDs 7, 9, and 11 (DEX3) or saline (n = 15; 7 females, 8 males) according to the same dosing schedule. Pups were placed back into their home cages with their respective dams following DEX or saline injections and were observed to determine if there were any obvious abnormalities in feeding behavior or maternal care. Body weights were obtained on PNDs 7, 9, 11, 14, 21, 28, 34, and 43. In Study 2 pups received the same saline (n =14; 9 females, 5 males) and DEX3 (n = 14; 7 females, 7 males) treatments that were used in Study 1. Body weights, however, were measured on PNDs 7, 9, 11, 12, 21, and 43. The concentrations of the preservative-free prodrug dexamethasone sodium phosphate USP (Voigt Global Distribution LLC, Lawrence, KS, USA), the water-soluble inorganic ester of dexamethasone typically used clinically, were expressed as molar equivalents to DEX. Dexamethasone was solubilized in 0.9% saline solution and administered at 10 μL of vehicle per gram weight of animal.

### 2.3. Behavioral Tests

#### 2.3.1. 1-h Locomotor Activity/Exploratory Behavior Test

Locomotor-related behaviors were evaluated in transparent polystyrene enclosures (47.6 × 25.4 × 20.6 cm) for 60 min, as previously described [21] using computerized photobeam instrumentation (MotorMonitor, Hamilton-Kinder, LLC, Poway, CA). General activity variables (total ambulations, number of rearings, and distance traveled in a peripheral zone) along with measures of emotionality (time spent, entries made, and distance traveled in the central zone) were quantified.

#### 2.3.2. Sensorimotor Battery

Balance, strength and coordination were evaluated by testing the mice on a battery of sensorimotor measures using previously described methods [21]. The battery included ledge and platform tests that involved determining how long a mouse could remain on an elevated, narrow (0.75 cm wide) Plexiglas ledge or on an elevated small circular wooden platform with rounded edges (1.0 cm thick; 3.0 cm diameter). A walking initiation test was also performed by measuring the time it took a mouse to move out of a small square (21 × 21 cm) outlined on a black tabletop, while general coordination and strength were studied through the use of the pole and inclined and inverted screen tests. The pole test involved placing a mouse “head up” with its forepaws on top of a finely textured rod (diameter 8 mm; height 55 cm) and timing how long it took the mouse to turn and climb down the pole. In the 90° inclined screen test, mice were placed in the middle of an elevated wire mesh grid inclined to 90° with their heads oriented down, and the time taken by the mouse to turn and climb to the top of the screen was determined. For the inverted screen test, a mouse was placed on the screen described above, which was inclined to 60°, and when the mouse appeared stable the screen was inverted to 180°. The time the mouse was able to remain hanging upside down on the screen before falling was determined. Two trials were administered for each test in the battery, and means were computed for each mouse.

#### 2.3.3. Rotarod

Motor coordination and balance were studied further by evaluating the mice on the rotarod (Economex, Columbus Instruments, Columbus, OH) test. The procedure was similar to previously described methods [22,23] where a test session included 3 consecutive conditions: 1 trial on a stationary rod (60 s maximum); 2 trials on a rotating rod with a constant speed (2.5 rpm for 60 s maximum); and 2 trials on a rod which had an accelerating rotational speed (2.5–10.5 rpm over 0–180 s). Motor learning was minimized by using 3 test sessions, each of which was separated by 4 days of no testing. Time spent on the rod in each condition was used as the dependent variable.

#### 2.3.4. Grip Strength

Forelimb grip strength was measured using a grip strength meter (Ugo Basile, Collegeville, PA). The grip strength procedure has been described in detail previously [23,24]. Briefly, a mouse was trained to grab and hold onto a metal trapeze-shaped grasping bar (3.0 cm in length; 2.0 mm in diameter) attached to a force transducer, when pulled by the tail (hindlimbs were unsupported). When the horizontal backwards pulling force overcame the grip strength of the mouse, its grasp was released and the peak pull force on the grasping bar was measured and stored by a peak preamplifier and presented on a digital display (in gram-force units). Each mouse received 3 days of habituation and grasping training which involved performing multiple trials until it was judged that a mouse responded with 5 robust, stable pulls. Grip strength testing occurred on the following 2 days when 5 trials involving stable responding were conducted.

### 2.4. Complex Activity Wheel Task

#### 2.4.1. Normal Activity Wheel

**Figure 1:**
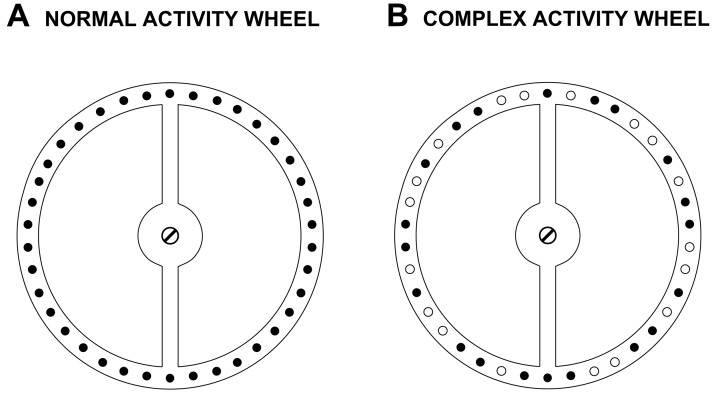
Schematic representation of normal and complex activity wheels. (**A**) Baseline activity was measured on a normal activity wheel that contained a full complement of 38 consecutive rungs. (**B**) Fine motor co-ordination was evaluated using a complex activity wheel that involved the use of a customized wheel in which 18 crossbars or rungs were removed at irregularly spaced intervals. The dark circles represent the presence of a rung and white circles represent a removed rung.

To acquire baseline voluntary wheel running activity data, the mice were tested on a normal mouse activity wheel (Mouse Motor Skill Sequences Activity Wheel, Lafayette Instrument, Lafayette, IN) for 1 h on 5 consecutive days. Each polycarbonate activity wheel chamber measured 31.5 × 19.5 × 19.5 cm, and contained a conventional anodized aluminum running wheel suspended from the chamber top, and a thin layer of bedding covered the chamber floor. An optical rotation sensor mounted 0.508 cm from the rungs transmitted wheel rotation information to the Activity Wheel Monitoring System Software (Lafayette Instrument, Lafayette, IN) on a nearby computer. The normal activity wheel contained 38 consecutive rungs (0.4 cm in diameter) that were spaced 0.614 cm apart (Figure 1). One revolution of the wheel equaled 0.40 meters in distance traveled. For each of 5 consecutive testing days, a mouse was placed in the activity wheel chamber and allowed to voluntarily run in the wheel for 1 h while the number of wheel rotations (counts) and distance traveled were quantified and computed by the system software across the 1-h testing session. In Study 1, mice received two “familiarization” sessions in the normal activity wheel before data collection was initiated, whereas the procedure for Study 2 did not include these 2 sessions and data were collected when mice were first exposed to the normal wheel.

In Study 1, the data were collected in 10-min bins to be consistent with a previously published method using the same instrumentation [25]. Although this allowed for a comparison of data across studies, the limited temporal resolution of the data reduction procedure did not allow for true running speed to be quantified. In order to calculate true running speeds and quantify other variables such as time spent not running, the data analysis for Study 2 included changing the data sampling interval to 10-s bins and the use of other additional specific data reduction procedures (see below). In Study 2, the average running speeds were calculated by computing the mean of all average speed values from 10-s bins within which at least 6 wheel rotations occurred (minimum speed = 14.4 m/min). This criterion was determined through observations of wheel running which showed that if a mouse executed 6 full rotations of the normal activity wheel, there could be no periods of time when wheel running was not occurring during a 10-s interval. The outcome variable, time spent not running in the wheel, was estimated by summing all 10-s intervals during which there were no wheel rotations. The maximum speed achieved during all 10-s intervals through the 1-h testing period also served as a dependent variable. Since the 10-min sampling interval was incompatible with the correct calculation for certain variables, distance traveled was the only variable analyzed from Study 1—wheel rotation counts yielded the same results—whereas distance traveled, average and maximum running speeds, and time spent not running in the wheel were analyzed from Study 2.

#### 2.4.2. Complex Activity Wheel

To provide a more sensitive test than that which is offered by the normal wheel for assessing fine motor coordination between the fore- and hind-limbs, the mice were tested on the complex activity wheel. This task involved the use of “customized” running wheels which included altering the normal wheels described above by removing 18 rungs thereby creating irregularly-spaced intervals between rungs that were either 0.614, 1.6, or 2.6 cm in distance according to the pattern shown (compare panels A and B in Figure 1). All other parameters of the activity wheel apparatus remained the same as in the normal activity wheel phase. Mice were tested on the complex wheel during each of 2 weeks (acquisition and performance test 1), then received a 6-week respite from testing, and were subsequently re-tested during each of 2 additional consecutive weeks (performance tests 2 and 3). On each test day a mouse was placed in the complex wheel chamber and allowed to voluntarily run in the wheel for 1 h while the data were collected in either 10-min (Study 1) or 10-s (Study 2) bins, and were analyzed from Studies 1 and 2 as described above for the normal activity wheel with one exception. The exception relates to the data reduction procedures that were used in Study 2 for calculating average running speed. Specifically, in Study 2, the average running speeds on the complex wheel were calculated by computing the mean of all average speed values from 10-s bins during which at least 4 wheel rotations occurred (minimum speed = 9.6 m/min). Further observations of wheel running showed that the slower running speeds exhibited in the complex wheel compared to those in the normal activity wheel necessitated lowering the data reduction criterion for computing average running speeds from 6 to 4 wheel rotations per 10-s bin.

#### 2.4.3. Immunohistochemistry

Brains from two sets of mice were used for histological analyses involving immunohistochemistry (IHC): 1) mice that were euthanized 4 h after a single exposure to DEX (n = 3) or saline (n = 3) on PND 7, and were prepared for activated caspase-3 (C3A) IHC; 2) mice that were euthanized after completion of behavioral testing in Study 2 on PND 190 that were prepared for NeuN and calbindin IHC. General IHC procedures similar to those previously described [8] included deeply anesthetizing and transcardially perfusing the mice with 4% paraformaldehyde fixative in 0.1 M TRIS buffer. After postfixation, the brains were removed and vibratome sectioned in the sagittal plane at 75 μm. For IHC, C3A (Cell Signalling Technology Inc, Danvers, MA), NeuN (Chemicon, Billerica, MA) and calbindin (Sigma-Aldrich, St. Louis, MO) primary antibodies were used to selectively label proliferating neural progenitor cells undergoing apoptosis, or cerebellar neurons in the granule cell layer, and Purkinje cell layer, respectively. Sections from the brains of mice perfused 4 h after injection were quenched in a methanol solution with 3% hydrogen peroxide for 15 min, placed in blocking solution (2% BSA, 0.2% milk, 0.1% Triton X-100 in PBS), and incubated in C3A (1:1000) primary polyclonal antibody overnight. The next morning, sections were incubated in goat anti-rabbit biotinylated secondary antibody (1:200), reacted with ABC reagents, and chromogen labeled with VIP reagent using products from Vector Laboratories (Burlingame, CA). Sections from the behaviorally tested mice were subjected to the same procedures except that they were incubated in both NeuN (1:100) and calbindin (1:1000) primary monoclonal antibodies overnight and a goat anti-mouse secondary antibody (1:200) was used. While the double-staining method used with sections from the behaviorally tested mice leaves both granule cells and Purkinje cells labeled in the same section, the dramatic difference in their size and shape allows these cell types to be easily distinguished from each other. Compared to labeling each neuron type on different sections, this method has the advantage of eliminating variability due to performing granule and Purkinje cell counts on different sections and saves processing time and supplies.

#### 2.4.5. Stereology

To stereologically assess neuronal counts in the behaviorally tested mice, sections were chosen in a systematic random sampling procedure and the interval, counting frame size, and distance between counting frames were adjusted so that a reasonable number (approximately 200) of cells were sampled. An unbiased estimate of the total number of immunoreactive NeuN granule cells and calbindin Purkinje cells was estimated in the same sections using the optical fractionator method (Stereoinvestigator Version 7.5, MicroBrightField Inc., Colchester, VT). Inter-litter variability was counterbalanced in two ways. Firstly, an equal number of each treatment group was represented in each litter. Secondly, data were normalized by taking the cell count for each mouse and dividing it by the average cell counts of the saline-treated mice from the same litter. In order to express this number as neuronal counts (rather than a dimensionless ratio), all numbers were then multiplied by the average of the cell counts for all saline-treated mice in every litter.

#### 2.4.6. Statistical Analyses

Analysis of variance (ANOVA) models were used to analyze the data. Typically, the statistical models included between-subjects variables of Group (DEX3 *vs.* saline controls) and Gender (male *vs.* female), and one within-subjects variable (e.g. days of testing). The Huynh Feldt adjustment of alpha levels was utilized for all within-subjects effects containing more than two levels to protect against violations of sphericity/compound symmetry assumptions. Bonferroni correction was used to help maintain alpha levels at 0.05 when multiple comparisons were conducted.

Several analyses were used to evaluate the complex activity wheel data. First, individual repeated measures ANOVAs (rmANOVAs) were used to analyze averaged weekly performances during the initial testing (acquisition and performance test 1) and re-testing (performance tests 2 and 3) phases to minimize complexity and alpha inflation. This ANOVA model involved 2 between-subjects variables: Group (saline *vs.* DEX3), and Gender (male *vs.* female), and 1 within-subjects variable, Test (acquisition *vs.* performance test 1, or performance test 2 *vs.* test 3). Follow-up ANOVAs were conducted for each week of testing following a significant effect of Group in the initial analyses. These follow-up ANOVAs included Group as a between-subjects variable and also Gender if this effect was significant during the initial analysis, while Test Days (1–5) served as a within-subjects variable.

## 3. Results

### 3.1. Body Weights

#### Study 1

**Figure 2:**
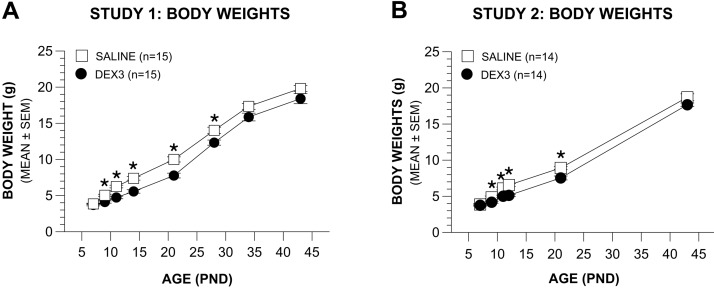
Body weights are reduced following DEX3 treatment. (**A**) In Study 1, DEX3 mice (n = 15; 6F, 9M) exhibited significantly reduced body weights compared to saline controls (n = 15; 7F, 8M) from PND 9 to PND 28 (* *p* < 0.0063). (**B**) In Study 2, DEX3 mice (n = 14, 7F, 7M) again exhibited significantly reduced body weights compared to saline controls (n = 14, 9F, 5M) from PND 9 through PND 21 (* *p* < 0.007).

Effects of the DEX3 treatment on body weights in Study 1 (Figure 2A) were analyzed by a rmANOVA containing the variables Group and Age (PNDs 7, 9, 11, 14, 21, 28, 34, 43). This analysis revealed significant effects of Group, [*F*(1,28) = 17.89, *p* = 0.0002], and Age, [*F*(7,196) = 908.10, *p* < 0.00005], showing that, in general, the DEX3 treatment significantly reduced body weight. Subsequent pair-wise comparisons indicated that the body weights of the DEX3 and saline control groups differed significantly (beyond Bonferroni correction: *p* < 0.0063) on PNDs 9, 11, 14, 21, and 28. In an effort to maintain sensitivity for detecting group differences and to simplify interpretation, the variable gender was not included in the original overall rmANOVA. To make sure that the omission of this variable did not provide a systematic bias, we re-ran the rmANOVA and included gender. As expected, we found a significant main effect of Gender, [*F*(1,26) = 5.16, *p* = 0.032], but importantly, the Group by Gender interaction was not significant (*p* > 0.10), thus confirming a lack of systematic bias resulting from omitting this variable. Although significant differences in absolute body weights between the groups were found up to PND 28, the DEX3 mice began to show healthy weight gains shortly after the end of the DEX3 treatment on PND 11 such that the weight gains from PND 11 to PND 14 (or thereafter) were found not to be significantly different between the two groups (*p* > 0.05).

#### Study 2

Differences in body weights across treatment and on into the juvenile period in Study 2 (Figure 2B) were similar to those found during Study 1. Specifically, a rmANOVA conducted on body weight data from PNDs 7, 9, 11, 12, 21, and 43 yielded significant effects of Group, [*F*(1,26) = 15.63, *p* = 0.0005], and Age, [*F*(5,130) = 1854.82, *p* < 0.00005], and a Group by Age interaction, [*F*(5,130) = 3,93, *p* > 0.039], showing that the DEX3 treatment significantly reduced body weights during and after treatment. Pair-wise comparisons indicated that the body weights of the two groups differed significantly on PNDs 9 through 21 (*p* < 0.007), although differences were no longer significant at PND 43. Similar to the analyses used in Study 1, we re-ran the overall rmANOVA for the Study 2 body weights and included gender as a variable, which resulted in the same outcomes. Namely, that there was a significant main effect of Gender, [*F*(1,24) = 4.45, *p* = 0.049], but the Group by Gender interaction was nonsignificant (*p* > 0.10).

### 3.2. 1-h Locomotor Activity/Exploratory Behavior Test

#### Study 1

**Figure 3:**
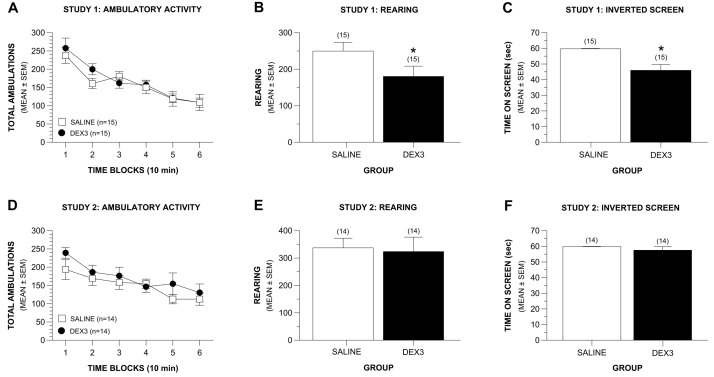
Early post-weaning performance of DEX3-treated and saline control mice on the 1-h locomotor activity/exploratory behavior test and battery of sensorimotor measures for Studies 1 and 2. (**A**) In Study 1, both groups exhibited similar levels of ambulatory activity as indexed by total ambulations (whole body movements) across the 60 min test. (**B**) However, the DEX3 mice demonstrated significantly less rearing than saline controls (* *p* = 0.044), and (**C**) also spent significantly less time hanging from the inverted screen in Study 1 compared to saline controls (*p = 0.001). For Study 1: DEX3 group - n = 15, 6F, 9M; saline control group - n = 15, 7F, 8M. (**D**) Similar to Study 1, no differences were observed between groups in ambulatory activity across the 60-min test in Study 2. However, in contrast to Study 1, no differences were observed between groups in rearing (**E**) or on the inverted screen test (**F**) in Study 2. For Study 2: DEX3 group - n = 14, 7F, 7M; saline control group - n = 14, 9F, 5M.

*The* results of a rmANOVA revealed that the groups in Study 1 exhibited similar levels of ambulatory activity across the 60-min test session (Figure 3A). Specifically, no significant differences were observed between groups in terms of total ambulations (whole body movements) nor were there any effects involving gender, and both groups showed similar degrees of habituation of activity across the test session (Figure 3A). Similarly, no Group or Gender effects were observed concerning the time spent, distance traveled, or entries made in the center of the test field or in the distance traveled in the peripheral zone of the field. However, the DEX3 group exhibited significantly less rearing during the test session, [Figure 3B; *F*(1,26) = 4.47, *p* = 0.044], suggesting possible hind-limb dysfunction, balance disturbances or alterations in exploratory behaviors. Again, no effects involving gender were found.

#### Study 2

Like the results from Study 1, a rmANOVA on total ambulations did not reveal any significant overall effects involving Group thus showing that the DEX3 and saline control mice did not differ in terms of their general ambulatory activity across the 60-min test session. Also, there were no significant differences between the groups with regard to the degree of habituation of activity that occurred during the test period (Figure 3D). Importantly, however, no differences were found between the DEX3 and saline mice in terms of rearing (Figure 3E), results which are in contrast with those from Study 1 which showed DEX3 mice had significantly lower levels of rearing compared to the saline controls. The two groups also did not differ in the distance traveled in the peripheral zone of the test field. No overall effects involving Gender were found with regard to these variables. In contrast to the findings from Study 1, analysis of the emotionality data did reveal consistent gender influences, however. For example, significant Group by Gender interactions were found for distance traveled, time spent, and entries made into the center of the test field, [*F* (1,24) = 6.81, *p* = 0.015; *F*(1,24) = 8.69, *p* = 0.007; *F*(1,24) = 5.78, *p* = 0.024, respectively]. Subsequent pair-wise comparisons showed that, compared to the saline control males, the DEX3 males traveled a significantly greater distance, spent significantly greater time, and made significantly more entries in the center compared to that of the saline controls (*p* = 0.011, 0.006, and 0.019, respectively; data not shown). The females of the two groups were never found to differ significantly on any of these three variables. Thus, the DEX3 males exhibited less anxiety-like behaviors as indexed by their willingness to go into the center of the test field compared to the saline control males.

### 3.3. Sensorimotor Battery, Rotarod, and Grip Strength

#### Study 1

Results from the sensorimotor battery in Study 1 showed that the DEX3 mice did not differ from the saline control group in terms of performance on the ledge or platform tests (data not shown) suggesting that the reduced rearing was probably not due to balance disturbances. The groups also did not exhibit performance differences on the walking initiation, pole, or 90° inclined screen tests (data not shown) suggesting that they did not differ with regard to movement initiation or speed during sensorimotor tasks. However, the DEX3 mice spent significantly less time on an inverted screen (Figure 3C) compared to the controls, [*F*(1,26) = 14.70, *p* = 0.001], suggesting impaired strength and/or co-ordination in the DEX3 mice. No effects involving gender were observed for the inverted screen data. No performance differences were found between groups on the stationary rod, constant speed rotarod, accelerating rotarod, nor on the forelimb grip strength test (data not shown).

#### Study 2

No differences were observed between the DEX3 and control mice on the ledge, platform, pole, and the inclined and inverted screen tests within the sensorimotor battery, although a significant Group by Gender interaction was found following the ANOVA on the walking initiation data, [*F*(1,24) = 5.28, *p* = 0.031]. Pair-wise comparisons showed that this effect was mainly due to the DEX3 females taking significantly less time to leave the square compared to the female saline control mice (*p* = 0.007) although the males from each group did not differ on this variable (data not shown). Note that the lack of differences found on the inverted screen test in Study 2 (Figure 3F) were in contrast to the results from Study 1, which showed that the DEX3 mice were significantly impaired on this test. However, consistent with findings from Study 1, we found no significant differences between the DEX3 and saline control groups on the rotarod or grip strength tests in Study 2. In Study 2 we also evaluated the mice on contact righting to determine if vestibular disturbances were likely present in the DEX3 mice which would affect their performance on the motor co-ordination tests, but no deficits were observed in the DEX3 mice on this test either (data not shown).

### 3.4. Normal and Complex Activity Wheels

#### 3.4.1. Distance Traveled Data

##### Study 1

**Figure 4:**
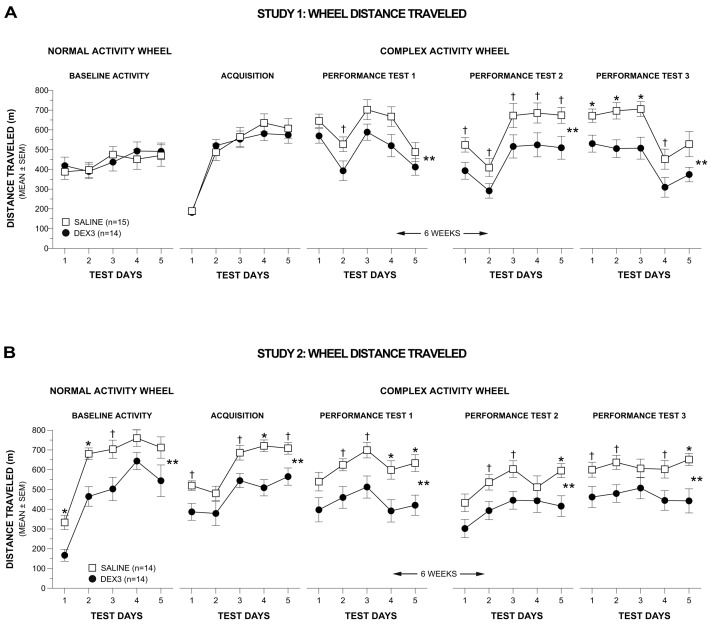
DEX3-treated mice exhibited performance deficits on the complex activity wheel in terms of distance traveled. (**A**) In Study 1, DEX3 (n = 14; 6F, 8M) and saline control mice (n = 15; 7F, 8M) traveled similar distances in the normal activity wheel (baseline) and during acquisition training in the complex wheel (2 left-most panels). During performance test 1 (middle panel), DEX3 mice traveled significantly shorter distances than the saline controls (** *p* = 0.045), with differences between groups being largest on test day 2 († *p* = 0.038). During performance test 2 (2^nd^ right-most panel), DEX3 mice continued to travel significantly shorter distances compared to saline controls (** *p* = 0.019) when re-tested after a 6-week layoff (†p < 0.043 for days 1–5). Wheel running distances were also significantly shorter for DEX3 mice during performance test 3 (far right panel) compared to the saline control group (** *p* = 0.004 – group effect; * *p* < 0.009 for days 1, 2, and 3; † *p* = 0.04 for day 4). (**B**) In contrast to the results of Study 1, the DEX3 mice in Study 2 (n = 14; 7F, 7M) traveled significantly shorter distances (** *p* = 0.008) compared to saline controls (n = 14; 9F, 5M) during the normal activity wheel baseline testing (far left panel: *p = 0.003 and 0.002 for days 1 and 2, respectively; †p = 0.022 for day 3). Similarly contrasting data were found during acquisition on the complex activity wheel (2^nd^ left-most panel), where DEX3 mice again traveled significantly shorter distances (** *p* = 0.003) compared to the saline control group (* *p* = 0.0003 for day 4; † *p* < 0.012 for days 1, 3, and 5). During performance test 1 (middle panel) in Study 2, DEX3 mice traveled significantly shorter distances (** *p* = 0.008) than saline controls (* *p* = 0.009 and 0.004 for days 4 and 5, respectively; † *p* < 0.015 for days 2 and 3). Graphs in the two right-most panels show that the DEX3 mice continued to travel significantly shorter distances relative to saline controls after a 6-week layoff from testing as shown for performance test 2 (** p = 0.018 - group effect; *p = 0.009 for day 5; † *p* < 0.027 for days 2 and 3) and performance test 3 (** *p* = 0.017 – group effect; *p = 0.005 for day 5; † *p* < 0.045 for days 1, 2, and 4).

Analysis of the normal activity wheel (distance traveled) data in Study 1 showed that the DEX3 and saline control mice exhibited comparable levels of wheel running during the measurement of baseline activity levels (Figure 4A, far left panel).

The results of a rmANOVA conducted on the weekly averages across the first two weeks of testing on the complex wheel (acquisition *vs.* performance test 1) in Study 1 yielded a significant Group by Test interaction [*F*(1,25) = 6.43, *p* = 0.018], but no other significant overall effects involving Group or Gender, indicating that the groups performed differently depending on whether it was during acquisition or performance test 1. To clarify this effect another rmANOVA model containing one within-subjects variable, Test Day (1–5), and one between-subjects variable, Group, was conducted on the acquisition data and on the performance test 1 data. During acquisition (Figure 4A, 2nd left-most panel), DEX3 mice performed indistinguishably from the saline controls and both groups displayed improved performance over the 5 days of testing [*F*(4,108) = 91.64, *p* < 0.0005]. In contrast, during performance test 1 (Figure 4A, middle panel), while there was still a significant effect of Test Day [*F*(4,108) = 19.33, *p* < 0.0005], the DEX3 mice traveled a significantly shorter distance on the complex wheel, on average across test days, compared to the saline control group [*F*(1,27) = 4.43, *p* = 0.045]. Subsequent pair-wise comparisons showed that differences between groups were greatest on test day 2 (*p* = 0.038).

To determine whether the DEX3 mice would show evidence of recovery from the complex wheel running deficits they exhibited during performance test 1, or whether this deficit represented chronic impairment, both groups of mice were re-tested on the complex wheel (performance tests 2 & 3) after experiencing a 6-week respite from testing. The rmANOVA conducted on the weekly averages for performance tests 2 and 3 in Study 1 revealed significant main effects of Group [*F*(1,25) = 8.89, *p* = 0.006] and Gender [*F*(1,25) = 6.16, *p* = 0.020], and a significant Gender by Test (performance tests 2 *vs.* test 3) interaction [*F*(1,25) = 4.42, *p* = 0.046], suggesting that performance on the complex wheel differed between groups although the effect may have been gender- and/or test-dependent. To clarify these effects, a rmANOVA containing two between-subjects variables, Group and Gender, and one within-subjects variable, Test Day, were conducted for each re-test (performance tests 2 and 3). Gender was included in this ANOVA model given the significant main effect and interaction effects involving this variable in the overall analysis involving weekly averages. The rmANOVA conducted on the performance test 2 data (Figure 4A, 2^nd^ right-most panel) showed significant main effects of Group [*F*(1,25) = 6.28, *p* = 0.019] and Gender [*F*(1,25) = 8.53, *p* = 0.007], indicating that the DEX3 mice were still impaired on the complex wheel task, although gender effects may be present. Subsequent pair-wise comparisons showed that large differences existed between groups on days 1–5 (*p* < 0.043). Additional contrasts indicated that, on average across test days, the DEX3-treated females traveled substantially shorter distances on the complex wheel compared to saline-treated females (*p* = 0.031) although these differences were not significant considering Bonferroni correction (*p* < 0.025), while the two male groups performed comparably (data not shown).

The rmANOVA on the performance test 3 data in Study 1 (Figure 4A, far right panel) showed a significant main effect of Group [*F*(1,25) = 9.94, *p* = 0.004] but no significant overall effects involving Gender, although the effect of Test Day was significant [*F*(4,100) = 26.88, *p* < 0.0005]. Pair-wise comparisons showed that the DEX3 mice traveled significantly shorter distances relative to the saline control mice on Test Days 1, 2, and 3 (*p* < 0.009), while large differences were also observed for Test Day 4 (*p* = 0.040). Thus, the results from re-testing the mice after a 6-week respite confirmed the performance deficits in the DEX3 mice that were originally observed during performance test 1, and suggest that the deficits in fine motor co-ordination resulting from multiple neonatal DEX treatments were likely permanent. A transient gender effect was observed in only one out of the four complex wheel test periods and, within that period (performance test 2), there was no compelling evidence of differences between groups within either sex.

##### Study 2

The distance traveled data from the normal baseline activity and complex wheel acquisition phases in Study 2 (Figure 4B, 2 left-most panels) were in contrast to the earlier findings on these test phases in Study 1. Specifically, a rmANOVA conducted on the distance traveled data during the normal wheel baseline (Figure 4B, far left panel) yielded significant main effects of Group, [*F*(1,24) = 8.47, *p* = 0.008], and Test Day, [*F*(4,96) = 53.24, *p* < 0.00005], but no other significant effects involving Group or Gender. Pair-wise comparisons showed that the DEX3 group traveled significantly shorter distances than control mice on Test Days 1 (*p* = 0.003) and *2* (*p* = 0.002), with large differences also being observed on Test Day 3 (*p* = 0.022). Distances traveled on the first test day of baseline testing were substantially lower than those from Study 1, which likely reflects the fact that “familiarization” sessions were not given in Study 2 but rather that data were recorded upon initial exposure to wheel running.

A rmANOVA conducted on the weekly averages for the distance data derived for acquisition and performance test 1 in Study 2 resulted in a significant effect of Group, [*F*(1,24) = 7.81, *p* = 0.010], but no other significant effects involving Group or Gender were found. Based on our findings from Study 1, we conducted planned individual analyses on the acquisition or performance test 1 data. Significant main effects of Group, *F*(1,26) = 10.58, *p* = 0.003], and Test Day, [*F*(4,104) = 29.63, *p* < 0.00005], were found for the acquisition data (Figure 4B, 2nd left-most panel) suggesting that, on average, the DEX3 mice traveled a significantly shorter distance in the complex wheel than the saline control mice and that running distances varied across test days. Subsequent pair-wise comparisons showed that the two groups differed significantly on Test Day 4 (*p* = 0.0003) while large differences were also observed on Test Days 1, 3, 5 (*p* < 0.012). Similar effects were found from analyzing the data from performance test 1 (Figure 4B, middle panel) where significant main effects of Group, [*F*(1,26) = 8.13, *p* = 0.008], and Test Day, [*F*(4,104) = 11.68, *p* < 0.00005], documented impaired performance on the part of the DEX3 mice and that distances varied across days. Pair-wise comparisons showed that significant differences between the DEX3 and saline control groups were found on Test Days 4 (*p* = 0.009) and 5 (*p* = 0.004) with large differences being present on Test Days 2 and 3 (*p* < 0.015).

Analysis of the distance data from performance tests 2 and 3 in Study 2 (Figure 4B, 2 right-most panels) documented that the DEX3 mice exhibited a continuing deficit in terms of distance traveled in the complex wheel when the mice were re-tested after a 6-week respite from wheel running. Specifically, a significant Group effect, [*F*(1,24) = 5.92, *p* = 0.023] was observed from the rmANOVA conducted on the weekly average distances for the performance tests 2 and 3 but no other significant effects involving Group or Gender were found. Subsequently, significant effects of Group, [*F*(1,26) = 6.44, *p* = 0.018], and Test Day, [*F*(4,104) = 7.30, *p* = 0.0003], were found following a rmANOVA on the performance test 2 data (Figure 4B, 2nd right-most panel) demonstrating the significant impairment in the DEX3 mice during this test period. Comparisons on daily performances showed that significant differences occurred on Test Day 5 (*p* = 0.009), although large differences also occurred on Test Days 2 and 3 (*p* < 0.027). The rmANOVA on the performance test 3 data (Figure 4B, far right panel) yielded a significant main effect of Group, [*F*(1,26) = 6.52, *p* = 0.017], thus showing the continuing deficits of the DEX3 mice on this variable. Pair-wise comparisons indicated that the group performances differed significantly on Test Day 5 (*p* = 0.005) with large differences also being found on Test Days 1, 2, and 4 (*p* < 0.045).

#### 3.4.2. Additional Wheel-Running Variables

In Study 2, the data were reduced into 10-s bins in order to accurately quantify other wheel running variables, which would provide insight into the nature of the decreased distances traveled by the DEX3 mice. The results of these analyses showed that the differences in wheel-running distances between the two groups on the normal and complex activity wheels were mostly due to differences in times spent not running in the wheels rather than to differences in running speeds.

#### 3.4.3. Time Spent Not Running

**Figure 5:**
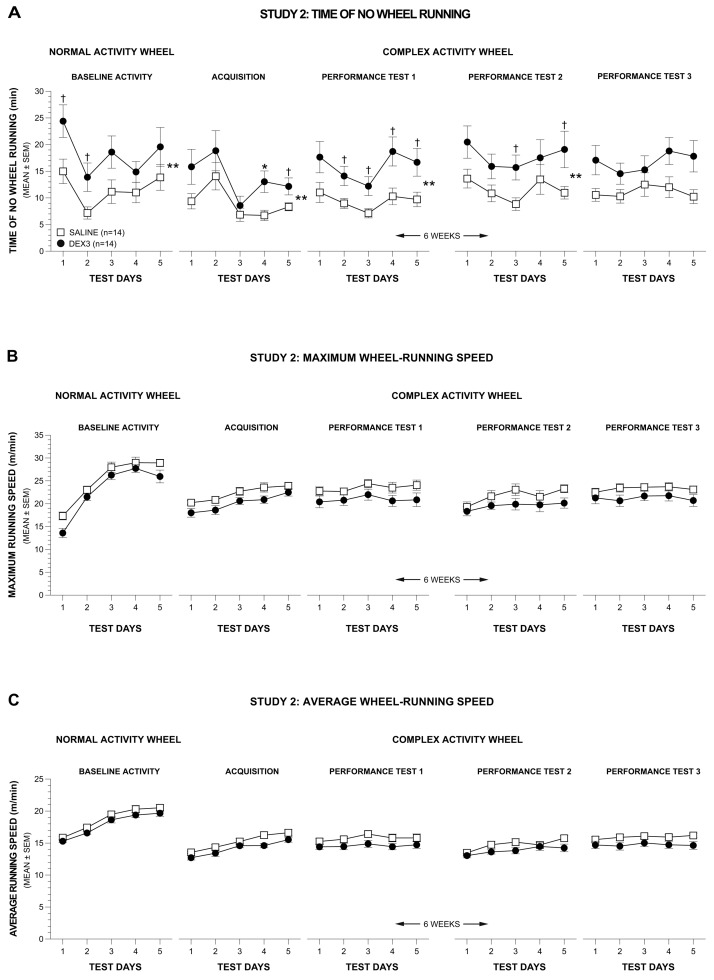
In Study 2, DEX3-treated mice spent more time not running in the activity wheels than saline controls, but exhibited similar maximum and average running speeds. (**A**) DEX3 mice spent significantly more time not running in the normal activity wheel during baseline measures (far left panel) compared to saline controls (** *p* = 0.034 – group effect; †*p* = 0.030 and 0.036 for days 1 and 2, respectively), as well as during acquisition training in the complex wheel (** *p* = 0.038 – group effect; * *p* = 0.009 for day 4; † *p* = 0.039 for day 5), and during performance test 1 (middle panel; ** *p* = 0.015 – group effect; † *p* < 0.026 for days 2–5). DEX3 mice continued to spend significantly more time not running on the complex wheel compared to saline control mice (** *p* = 0.027) when retested during performance test 2 (2nd right-most panel) after a 6 week layoff († *p* < 0.034 for days 3 and 5). However, unlike the results from the previous complex activity wheel tests, an ANOVA on the performance test 3 data yielded a nonsignificant Group effect (right panel). Other data that were in sharp contrast to the other wheel-running variables included a lack of significant differences between groups for the maximum wheel-running speed (**B**) and the average wheel-running speed (**C**) that were measured during testing on both the normal or complex activity wheels. For all graphs: DEX3 group - n = 14; 7F, 7M and saline control group - n = 14; 9F, 5M.

A rmANOVA conducted on the data pertaining to time spent not running during the normal wheel baseline period produced significant effects of Group, [*F*(1,24) = 5.05, *p* = 0.034], and Test Day, [*F*(4,96) = 8.27, *p* = 0.0001], but no other effects involving Group or Gender (Figure 5A, far left panel), thus showing that the DEX3 mice spent significantly more time not running in the wheel relative to the control group, and that non-running in the wheel times varied across days. Pair-wise comparisons indicated that differences between groups were greatest on Test Days 1 and 2 (*p* = 0.030 and 0.036, respectively).

The rmANOVA on the weekly averages from acquisition and performance test 1 resulted in a significant main effect of Group, [*F*(1,24) = 5.44, *p* = 0.028], but no other significant overall effects involving Group or Gender were found. The individual rmANOVA on the acquisition data also yielded significant effects of Group, [*F*(1,26) = 4.77, *p* = 0.038], and Test Day, [*F*(4,104) = 7.40, *p* = 0.0004], although no other significant overall effects involving Group were observed (Figure 5A, 2nd left-most panel). Compared to the saline controls, the DEX3 mice spent significantly less time running in the complex wheels on Test Day 4 (*p* = 0.009) while large differences were also observed on Test Day 5 (*p* = 0.039). Similar results were found from the rmANOVA on the data from performance test 1 (Figure 5A, middle panel) which showed significant effects of Group, [*F*(1,26) = 6.84, *p* = 0.015], and Test Day, [*F*(4,104) = 7.96, *p* < 0.00005]. Pair-wise comparisons showed that there were large group differences in times spent not running in the complex wheels on Days 2–5 (*p* < 0.026).

Analysis of the data from performance tests 2 and 3 (Figure 5A, 2 right-most panels) showed similar differences between the groups with the DEX3 mice spending less time running in the complex wheels compared to the saline control group, although there was less variation in performance across test days compared to the acquisition and performance test 1 data. Specifically, the rmANOVA on the weekly averages from performance tests 2 and 3 yielded a significant main effect of Group, [*F*(1,24) = 5.13, *p* = 0.033], but no other significant effects of Group or Gender were found. The individual rmANOVA on the data from performance test 2 (Figure 5A, 2nd right-most panel) revealed a significant effect of Group, [*F*(1,26) = 5.51, *p* = 0.027] with large differences in times spent not running in the complex wheels being found for Test Days 3, and 5 (*p* < 0.034). The individual rmANOVA on the data from performance test 3 (Figure 5A, far right panel) revealed no significant effects involving Group. The effect of Test Day was not significant for either of the two individual performance tests.

#### 3.4.4. Maximum and Average Running Speeds

In contrast to the data pertaining to time spent not running in the wheel, analysis of the maximum and average wheel-running speeds did not reveal reliable differences between groups (Figure 5B and Figure 5C). Specifically, ANOVAs conducted on the maximum or average speed data from the normal wheel baseline testing did not result in any overall effects involving Group. Similarly, none of the ANOVAs conducted on the weekly averages (either acquisition *vs.* performance test 1 or performance test 2 *vs.* performance test 3) yielded any significant overall effects involving Group. Thus, no individual ANOVAs were conducted on either the acquisition data or on the data from any of the single performance tests. Based on the analyses that included weekly averages for initial testing (acquisition and performance test 1) and re-testing after a 6-week respite (performance tests 2 and 3), we conclude that there was no reliable evidence showing that either the maximum or average speeds differed between the two groups.

#### 3.4.5. Acute and Long-Term Histopathological Effects of Neonatal DEX

**Figure 6:**
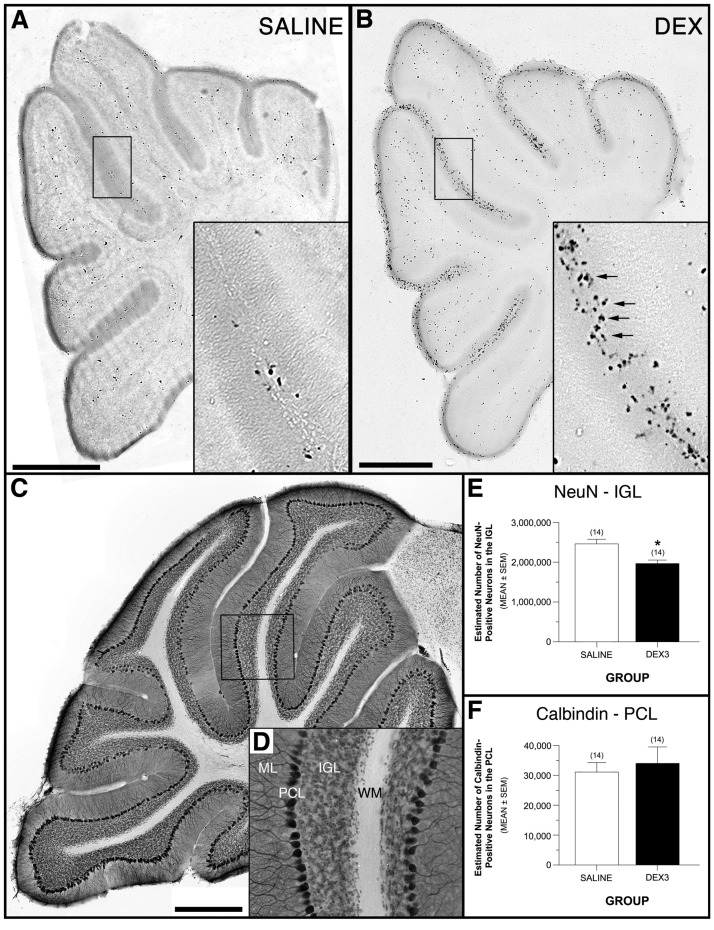
Histopathological effects of neonatal DEX exposure in mice. (**A-B**) Representative sagittal sections of the cerebellum are presented showing activated caspase-3 immunoreactivity from postnatal day 7 (PND 7) mouse pups that were treated with either saline (A) or a single dose of DEX (3.0 mg/kg) (B) and then processed for immunohistochemistry 4 h later. Note that DEX exposure (B) produced robust increases in the number of caspase-3 positive profiles in the outermost layer (external granule layer) of the cerebellum, indicating a dramatic elevation in the level of apoptotic cell death of neural progenitor cells compared to that observed in saline control mice (A). The smaller boxed regions in (A) and (B) indicate magnified areas represented in the larger boxed insets in the lower right portion of each panel where arrows indicate activated caspase-3 profiles. (**C-D**) A representative sagittal section is shown from the cerebellum of a 190-day old mouse that had received DEX (3.0 mg/kg) on PNDs 7, 9, and 11 (DEX3 treatment) and completed behavioral testing in Study 2. Sections were double stained for NeuN, which selectively stains granule cell neurons within the internal granule cell layer (IGL), and calbindin, which selectively labels Purkinje cells within the Purkinje cell layer (PCL). A higher magnification of the boxed region of “C” is shown in “D” where positive NeuN staining may be observed in the IGL and positive calbindin profiles may be seen in the PCL. (**E-F**) Stereologically derived neuronal counts of NeuN-positive profiles were significantly decreased in DEX3 mice (* *p* = 0.0013) relative to saline controls (E), although calbindin-positive profiles were not different between the two groups (F). ML = molecular layer. WM = white matter. Scale bar = 500 μm.

Treating mice with a single dose of DEX on PND 7 resulted in a robust increase in C3A-positive cellular profiles in the outer cerebellar EGL compared to saline control mice (Figure 6A-B). These findings are consistent with the results from our previous study in which we demonstrated that this DEX treatment leads to significant increases in the number of C3A-positive profiles, and that these C3A immunoreactive cells are neural progenitor cells that undergo neuroapoptotic cell death [8].

Figure 6C-D show the distribution of NeuN- and calbindin-positive profiles from the cerebellar IGL and Purkinje cell layer, respectively, in a representative, double-labeled section from a behaviorally tested DEX3 mouse. An ANOVA on the NeuN-positive cell data yielded a significant effect of Group, [*F*(1,24) = 13.32, *p* = 0.0013], while the effects of Gender and Group by Gender interactions were not significant, thus documenting that DEX3 treatment resulted in a significant reduction (20%) in neurons from the IGL (Figure 6E). These results suggest the apoptotic death of EGL NPCs results in a decrease in the number of IGL granule cells produced. In contrast, the ANOVA conducted on the calbindin-positive Purkinje cell counts did not reveal any significant effects involving Group, thus showing that the DEX3 treatment did not result in any discernible loss of Purkinje cells (Figure 6F). This result shows that DEX exposure has no significant effect on the neurons in the adjacent Purkinje cell layer, which are produced by a different proliferative layer prior to birth.

## 4. Discussion

Human preterm infants with chronic lung disease have frequently been treated with GCs such as DEX. For example, a review of recent randomized controlled trials to evaluate the efficacy of GCs to prevent or treat bronchopulmonary dysplasia [15], reveals that such studies often involved exposing neonates to daily doses of DEX tapered over 7 to 42 days. As Watterberg *et al.*, [15] have reported, there is some evidence that multiple lower doses of DEX may have minimal side effects while still maintaining efficacy to treat respiratory dysfunction, although long-term follow-up studies are needed to adequately evaluate this. Thus, it is important to establish animal models to study the neurotoxic side effects of multiple exposures to GCs in a systematic and controlled manner.

The results from the present study demonstrate that, while a single PND 7 exposure to DEX (3.0 mg/kg) in the mouse produces increases in C3A-positive profiles in the neonatal EGL of the cerebellum, three exposures of DEX during the neonatal period (PNDs 7, 9, 11) results in permanent neuronal loss in the adult IGL that is associated with long-term, possibly chronic, behavioral deficits. The present results are consistent with our previous findings that a single dose of DEX at 3.0 mg/kg produces selective neural progenitor cell loss in the neonatal EGL that is apoptotic in nature and consequent permanent reductions in neuronal counts in the adult IGL [8]. In the present study, we chose to use the single PND 7 exposure to DEX for illustrating the acute apoptotic effects in the neonatal EGL since it better represents the distribution of C3A-positive profiles compared to the staining that would have resulted from processing the tissue after the third DEX exposure. Because cerebellar NPCs undergoing apoptosis are only C3A positive for several hours, the number of C3A-positive profiles would have been relatively sparse after the third DEX exposure due to the substantial attrition that would have occurred from the previous DEX administrations. In the present work, we also conducted extensive behavioral studies on mice exposed to the DEX3 treatment and found that it was associated with reliable deficits on the complex activity wheel test, which was used to assess fine motor co-ordination skills. We have also presented data, which suggest the possible nature of these deficits.

If a similar vulnerability exists in the human, clinical exposure to perinatal glucocorticoid therapy may produce behavioral deficits that are associated with disruptions in cerebellar development. This finding is consistent with clinical research showing that neonatal GC therapy is correlated with a 20.6% decrease in cerebellar volume (the most stunted region examined) when compared to untreated cohorts [26]. It also may explain why the neuromotor and cognitive deficits resulting from cerebellar dysfunction [27] can be similar to deficits seen in neonates exposed to GCs [3].

The DEX3 dosing regimen used in the present study reflected two main concerns. First, we wanted to model in mice the behavioral effects observed after multiple exposures in human neonates, and secondly, to limit growth retardation that may occur in mouse pups when they are exposed chronically to DEX since the presence of growth retardation could cloud the interpretation of the data. The DEX3 dosing regimen in the present study is different from one that was used in our recently published work [9], where we had given DEX daily on PND 0–7, which is during the early part of the apoptosis vulnerability window. The Noguchi *et al.*, 2011 study was begun after the present one and the former was designed to mimic the dosing approach used by another research group and was not concerned with growth retardation or modeling behavioral effects. Several considerations were used in choosing the time points for exposure for the present study. Firstly, the PND 4 mouse cerebellum corresponds to 20 weeks in gestation for a human [8]. Since glucocorticoid therapy is not recommended prior to 24 weeks in gestation [5], any time point at or before PND 4 would be inappropriate. Secondly, early (*i.e.*, prenatal) glucocorticoid therapy does not seem to produce the neuromotor and cognitive deficits seen with late (*i.e.*, postnatal) glucocorticoid therapy, which is often given chronically [2,3,5]. Based on this knowledge we decided to first expose animals on PND 7, which is equivalent to approximately 31 weeks of gestation in humans. Finally, since chronic exposure to DEX is associated with generalized growth retardation, we attempted to minimize the contribution of growth retardation to this effect by giving DEX every other day. The fact that body weights in the DEX3 group became similar to that of control mice by PND 34 or PND 43 (in Studies 1 and 2, respectively) indicates that this dosing approach was at least partially successful. However, we realize that our triple DEX exposure could still be producing some pathological changes in the brain or periphery that have not yet been detected and which may contribute to the behavioral deficit we have described here. Other approaches to producing apoptosis in cerebellar NPCs that do not also stimulate glucocorticoid receptors would need to be developed in order to disentangle the effects of chronic glucocorticoid stimulation on growth from that on NPC apoptosis. Regardless of this possibility, we feel that the cerebellar pathology and behavioral deficits produced by GC exposure in the mouse still provide a useful animal model for studying the iatrogenic effects produced clinically after perinatal GC therapy.

Our findings that DEX3 mice exhibit impaired performance on the complex activity wheel should be considered a reliable finding since deficits on the task were replicated within a given study across multiple performance tests as well as across two studies involving independent cohorts of mice. The results of each of the two studies indicate that DEX3 mice exhibited performance impairments on the complex wheel whether they showed deficits on a normal activity wheel (Study 2) or not (Study 1). In Study 1, DEX3 mice performed similarly to saline controls during baseline testing in a normal activity wheel and during the acquisition phase of the complex wheel task. However, reliable deficits were demonstrated during performance test 1, as well during performance tests 2 and 3, which were conducted 6 weeks after performance test 1. In contrast, the DEX3 mice in Study 2 showed impaired performance during baseline testing on the normal activity wheel as well as during acquisition and all performance test phases. Although the findings in Study 2 suggest that there may have been differences in general activity levels which may account for the differences on the complex activity wheel task, this seems unlikely since no differences were observed on the 1-h spontaneous locomotor activity/exploration task in either of the two studies when the mice were tested earlier in their lives. Moreover, as stated above, the DEX3 mice in Study 1 exhibited deficits during the performance tests on the complex wheel although they performed similarly to controls on the normal activity wheel during baseline testing.

Our original findings from Study 1 that DEX3 mice demonstrated impaired complex wheel performance were based on one variable, distance traveled. Although reference to this variable showed that some type of deficit was present in DEX3 mice on this task, it was not clear what aspects of behavior were compromised in these mice. By analyzing other variables in Study 2 we were able to provide greater insight into the nature of the behavioral deficit. Specifically, although the DEX3 mice traveled significantly shorter distances on the complex wheel, they did not differ significantly from saline controls in terms of either maximum or average running speed. However, analysis of another dependent variable, time spent not running in the complex wheel, revealed consistently shorter wheel-running times for the DEX3 group compared to the control mice. These findings suggest to us that the DEX3 mice were capable of running at speeds that were similar to those of controls but that subtle sensorimotor deficits directly resulted in the mice being unable to maintain control-like running speeds for long periods of time. An alternative, but related, hypothesis is that running in the complex wheel was more difficult for the DEX3 mice and therefore, it may not have been as reinforcing as it was for the saline controls. Thus, motivational abnormalities resulting from sensorimotor deficits also may underlie the significantly decreased running distances/times in the DEX3 mice. With regard to the maximum and average speed data, it is worth noting that the control mice displayed higher mean values on all of the fifty data points derived for the maximum and average speed variables even though statistically significant differences were not found between the two groups on either variable. This suggests to us that, although our assessment of complex-wheel running provides a more sensitive test than most other sensorimotor measures, additional components of behavioral analysis may be warranted to further enhance test sensitivity for evaluating possible deficits in the DEX3 mice on these two variables. For example, the inclusion of high-resolution video analysis during wheel running may provide for a more accurate characterization of the sensorimotor deficits in DEX3 mice as well as a better understanding of the nature of the impairments.

A potentially confounding influence on our interpretation of the activity wheel performance impairments is the finding that the DEX3 treatment significantly reduced body weights up until PND 34. However, our analysis of body weight gains showed that the DEX3 mice resumed normal body weight gains sometime between 1 to 3 days following the DEX treatment. Thus, there may have been some transient effect of disturbed nutritional status of the DEX3 mice that contributed to their behavioral impairments. A somewhat related potential confounding of our behavioral results is the possible smaller size of the DEX3 mice. The DEX3 mice appeared to be of a smaller size up until the latter stages of the juvenile period when body weights became normalized, but they did not appear to be smaller than saline control mice during wheel-running experiments, which were conducted during adulthood (*i.e.*, PNDs 88 and 75 for Studies 1 and 2, respectively). However, measurements of relevant body parts (e.g., trunk or limbs) of the mice were not performed to document this. Thus, it is possible that decreased size could have had an impact on complex wheel performance, although some of our results suggest this is not likely. For example, it does not seem likely that the decreased size of the DEX3 mice would significantly affect the distance traveled without affecting maximum or average running speeds. In addition, it is unclear how the reduced size of the DEX3 mice could explain their deficits on the normal activity wheel where the distances between rungs are consistently small and within easy reach. Future studies should include measurement of relevant body parts and the high resolution video analysis mentioned above to help determine if adult DEX3 mice have stunted growth and if this significantly contributes to the impaired performance on the complex wheel task or whether there are other aspects concerning the co-ordination of the fore- and hind-limbs which play important roles in the deficits.

Our results suggest that multiple neonatal DEX exposures produce subtle but demonstrable deficits on a task that requires fine motor control, and that gender does not generally interact with the DEX3 treatment in determining the magnitude of the complex wheel deficits. However, the results from the 1-h locomotor activity test in Study 2 suggest that gender may interact with the DEX3 treatment in producing alterations in emotionality, although this effect was not found in Study 1. Future studies should be conducted to evaluate more carefully how DEX treatment may interact with gender in affecting changes in emotionality.

Although we were able to demonstrate reliable performance impairments in DEX3 mice on the complex wheel test, no deficits were observed on the rotarod test nor were there any measures within the sensorimotor battery which consistently differentiated between the DEX3 and control mice. This is noteworthy since several of these assessments are often used to evaluate sensorimotor disturbances resulting from cerebellar dysfunction. The lack of robust sensorimotor impairments following neuronal loss in the IGL is consistent with other studies in mutant mouse models that involve similar IGL abnormalities. For example, the Ts65Dn mutant mouse which is segmentally trisomic for a region of mouse chromosome 16 and which has been used as a mouse model of Down syndrome, shows significant reductions in the volumes of both the IGL and molecular layer of the cerebellum as well as reduced cell density in the IGL, but motor deficits were not found to be associated with these abnormal neuropathological features [28]. More recently it has been reported that mice deficient in the nuclear orphan receptor TAK1, which is highly expressed in cerebellar granule cells, showed a significantly reduced width of the IGL, and an apparent delay in granule cell migration as well as a permanent deficit in foliation of lobules VI-VII. However, no impairments were observed on sensorimotor tests designed to assess gross motor co-ordination, strength, and position orientation (inverted screen, negative geotaxis, rotarod tests) or locomotor activity [29]. An interesting exception was the finding that TAK1-/- mice were impaired on indices of normal wheel running, particularly during the dark phase of the light-dark cycle when running is maximal.

Based on our own data and that of others described above, it appears that functional impairments following developmental disruptions in IGL morphology and neuronal density are not easily detected by many standard behavioral tests designed to assess motor/sensorimotor function. An exception to this “rule” appears to be the complex activity wheel task. Specifically, we have shown in the present study that our modified version of the complex activity wheel provides a sensitive test for detecting DEX3-induced performance deficits on a task that requires fine motor co-ordination of the fore- and hind-limbs. Our modified version of the complex activity wheel task is different from the motor skill sequence procedure described by Liebetanz and colleagues [20]—which also affords a high degree of test sensitivity—in that our protocol involves 1-h daily test sessions rather than a residential wheel that is available on a 24-h basis. It is our aim to use the complex activity wheel task in combination with the already-described additional behavioral analyses and control procedures to study further the effects of GCs on functional outcomes and to possibly determine whether there are adjuvant treatments that may enable GCs to be used more safely without inducing unwanted neurological side effects.

## 5. Conclusions

Multiple neonatal DEX exposures in the mouse produce permanent neuronal loss in the adult IGL of the cerebellum that is associated with long-term deficits in fine motor co-ordination. The present results further establish our mouse model involving neonatal GC exposure as a useful tool for studying the iatrogenic effects produced clinically following perinatal GC therapy in humans.
